# Signaling in the Physiology and Pathophysiology of Pancreatic Stellate Cells – *a* Brief Review of Recent Advances

**DOI:** 10.3389/fphys.2020.00078

**Published:** 2020-02-14

**Authors:** Agnieszka A. Kusiak, Mateusz D. Szopa, Monika A. Jakubowska, Pawel E. Ferdek

**Affiliations:** ^1^Faculty of Biochemistry, Biophysics and Biotechnology, Jagiellonian University, Kraków, Poland; ^2^Malopolska Centre of Biotechnology, Jagiellonian University, Kraków, Poland

**Keywords:** calcium, Hippo, pancreatic cancer, pancreatic stellate cells, pancreas, PDAC, signaling, Wnt

## Abstract

The interest in pancreatic stellate cells (PSCs) has been steadily growing over the past two decades due mainly to the central role these cells have in the desmoplastic reaction associated with diseases of the pancreas, such as pancreatitis or pancreatic cancer. In recent years, the scientific community has devoted substantial efforts to understanding the signaling pathways that govern PSC activation and interactions with neoplastic cells. This mini review aims to summarize some very recent findings on signaling in PSCs and highlight their impact to the field.

## Introduction

Since their discovery, pancreatic stellate cells (PSCs) have often been overlooked in favor of other cellular components of the pancreas – pancreatic acinar cells (PACs), ductal cells, and pancreatic islets – that all are present in evident abundance in the tissue architecture and perform obvious exocrine or endocrine functions. Only relatively recently have PSCs gained substantial attention from the scientific community, and this was once it became clear that these cells play important roles in pancreatic pathophysiology. PSCs occupy periacinar space and form a dynamic network in between pancreatic acini. In health, PSCs predominantly occur in a quiescent phenotype and are a minority among cellular components of the pancreas, comprising merely 4–7% of all cells in the organ (Apte et al., 1998). One characteristic propensity of quiescent PSCs is the presence of retinoid droplets in the cytosol (Apte et al., 1998). Upon activation triggered by tissue injury, PSCs undergo a series of morphological alterations, which include the loss of retinoid droplets, increased prominence of the ER network, and elongation of the cellular processes; they also start expressing alpha smooth muscle actin (α-SMA) as well as collagen types I and III, laminin, and fibronectin (Apte et al., 1998; Bachem et al., 1998). As a result, activated PSCs increase in numbers, and their products – extracellular matrix (ECM) components – may become a significant part of the organ. If unbalanced, this mechanism underlies the development of pancreatic fibrosis. This mini review aims to summarize the most recent studies on signaling in PSCs relevant in physiology and pathophysiology of the pancreas.

## Signaling and PSC Activation

**FIGURE 1:**
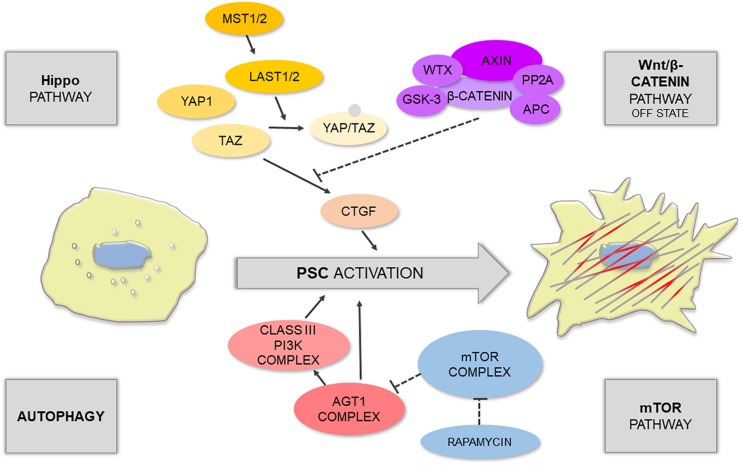
Schematic illustration shows that PSC activation is controlled by different signaling pathways.

Physiologically, the transition of quiescent PSCs into a proliferative, fibrogenic phenotype is an autonomous repair reaction to tissue injury. Since the damage to enzyme-storing PACs is particularly threatening to the integrity of the tissue, there is an obvious need for an efficient and well-balanced system orchestrating regeneration and containment of the injury with PSC activation at its center. This mechanism is expected to have cross-talks between numerous signaling pathways (Figure 1), given that PSC activation may be triggered by various stimuli, such as inflammatory mediators (Mews et al., 2002), alcohol metabolites (Apte et al., 2000), and growth factors, including transforming growth factors TGF-α and TGF-β, platelet-derived growth factor (PDGF) (Apte et al., 1999), or connective tissue growth factor (CTGF) (Gao and Brigstock, 2005). These activating factors are secreted not only by infiltrating immune cells but might also be coming from other sources e.g., acinar cells (PACs) (Masamune and Shimosegawa, 2009). Other pathophysiologically relevant factors associated with PSC activation include hyperglycemia (Nomiyama et al., 2007), hypoxia (Masamune et al., 2008), and oxidative stress (Casini et al., 2000). It is widely accepted that signaling pathways, such as the MAPK/ERK (Jaster et al., 2002; Yoshida et al., 2004), PI3K (McCarroll et al., 2004), Smad (Ohnishi et al., 2004), Jak/STAT (Komar et al., 2017), PKC (Nomiyama et al., 2007), and Hedgehog (Li et al., 2014), play a crucial role in PSC physiology and have been subjects of previous reviews (Masamune and Shimosegawa, 2009). However, recent evidence has indicated that the Hippo and Wnt pathways as well as authophagy and calcium signaling might be equally important players in PSCs. These new findings are briefly described below.

### Hippo Signaling Pathway

The Hippo signaling pathway is one of the main restrictors of cell proliferation that tend to promote apoptosis; it can be triggered by numerous microenvironmental cues, such as cell–cell contact, mechanotransduction, and cellular stress (Meng et al., 2016). During tissue repair, which normally requires cell proliferation, the Hippo pathway is often downregulated by phosphorylation of its main effectors: yes-associated protein 1 (YAP1) and transcriptional co-activator with PDZ-binding motif (TAZ) (Furth et al., 2018). This results in the switched off expression of YAP1/TAZ target genes, particularly CTGF, responsible for PSC activation. Liu J. et al. (2019) have recently demonstrated that acinar-specific deletion of central kinases in Hippo signaling – large tumor suppressor 1 and 2 (LATS1/2) – contributed to inflammation and severe fibrosis *in vivo*, even without the initiation of acinar cell rupture; whereas removal of CTGF with a neutralizing antibody attenuated this effect. The authors have also shown that in the mouse model of caerulein-induced pancreatitis there was an upregulation of secreted phosphoprotein 1 (SPP1) (Liu J. et al., 2019), a previously reported target of YAP1/TAZ involved in fibroinflammatory responses in other organs (Pardo et al., 2005). Interestingly, YAP1 and TAZ activity can be further modulated by the Wnt/β-catenin pathway. For instance, Wnt signaling can activate YAP1/TAZ by preventing their degradation by the β-catenin destruction complex (Hansen et al., 2015). Since the Wnt/β-catenin pathway plays a role in embryonic development as well as in homeostasis of the developed tissues, Wnt signaling becomes particularly important in the pathogenesis of proliferative diseases, including cancer.

### Wnt Signaling Pathway

The main factors associated with the Wnt signaling pathway were found both in the endocrine and exocrine pancreas; this includes β-catenin, Wnt2, Wnt5a, and Wnt inhibitors e.g., the secreted frizzled-related protein (SFRP) family (Heller et al., 2003). The distribution of these factors is dependent on the condition of the tissue – that is it changes upon tissue damage and in fibrosis. An analysis of human samples revealed that in the non-fibrotic pancreatic tissue, β-catenin is mainly located at the cell membrane of PACs, inhibitory SFRP4 is present in these cells, and Wnt2 is expressed at a low level (Blauer et al., 2019). In moderate fibrosis, the expression of Wnt2 in PACs increases, and β-catenin can now be found in the acinar nuclei indicating transcriptional activation; whereas in advanced fibrosis, β-catenin mostly localizes to PSCs (Blauer et al., 2019). Interestingly, this distribution pattern can be modeled *in vitro*. PSCs express β-catenin in monocultures, but when co-cultured with PACs, the levels of SFRP4 increase compared to monoculture, and thus PSCs do not exhibit nuclear distribution of β-catenin (Blauer et al., 2019). The above indicates that, in terms of Wnt signaling, a monoculture of PSCs reflects advanced stages of fibrosis, whereas a co-culture of PACs-PSCs has the expression pattern seen in moderate fibrosis. In another study, reversion of activated PSCs to quiescence was associated with upregulation of retinoic acid receptor β (RARβ), an increase in SFRP4 expression, and a reduction in nuclear β-catenin content in these cells (Carapuca et al., 2016). However, other reports suggest that both Wnt2 and SFRP4 might be elevated upon PSC activation (Hu et al., 2014). It is likely that PSCs could utilize multiple mechanisms regulating SFRP4, and thus its role in the interplay between PSCs and PACs, as well as in the process of PSC phenotype transition, is yet to be fully elucidated.

### Autophagy

Autophagy is traditionally viewed either as a means for nutrient seeking under stress or as a physiological process for a cell quality check and removal of dysfunctional cellular components (Glick et al., 2010; Bootman et al., 2018). While it is normally pro-survival, disruption of autophagy has been associated with non-apoptotic cell death via the interaction of its main effector Beclin-1 with anti-apoptotic Bcl-2 protein (Pattingre et al., 2005). Just like other autophagy-related proteins (Atg), including Atg1 or Atg5, Beclin-1 acts as an initiator of autophagosome formation (Shibutani et al., 2015). The relationship between PSC activation, pancreatic fibrosis, and autophagy has recently been well documented in the literature; autophagy has been suggested to be a source of energy and molecular material for PSC phenotype transition and ECM deposition (Endo et al., 2017). In highly fibrotic pancreatic solid tumors, cancer cells can stimulate autophagy of PSCs. This leads to the release of non-essential amino acids (NEAA), mainly alanine, from PSCs; NEAA are then used by cancer cells as an alternative (to glucose) energy source (Sousa et al., 2016). However, mouse models revealed that deregulation of Atg5 in the pancreas results in a similar phenotype to that seen in human chronic pancreatitis, indicating that the development of this disease appears to involve defects in autophagy (Diakopoulos et al., 2015). Mammalian target of rapamycin (mTOR) is a very well-known suppressor of autophagy that inhibits this process by regulating the activity of the initiator complex Atg1/ULK1 (Mizushima, 2010). In turn, mTOR can be upregulated by sphingosine-1-phosphate (S1P) or its analog, the immunomodulator fingolimod (FTY720), both of which inhibit autophagy (Thangada et al., 2014). FTY720 has been shown to suppress PSC activation and autophagy via the mTOR pathway; it can also increase the Bax/Bcl-2 ratio with a simultaneous decrease in the mitochondrial membrane potential (Cui et al., 2019). The mTOR pathway also links autophagy with intracellular calcium signals (Bootman et al., 2018).

### Calcium Signaling

Calcium signaling is one of the most universal pathways, regulating virtually every cellular process from excitability and motility to apoptosis (Clapham, 2007). Sophisticated machinery consisting of pumps, ion channels, and active transporters controls Ca^2+^ homeostasis; while spatiotemporal changes in Ca^2+^ concentration encode signals that exert cell responses. In the pancreas, Ca^2+^ signals play a particularly important role in regulating secretion of digestive enzymes by PACs, which has been a relatively frequent subject of research in the past decades (Petersen and Tepikin, 2008). In contrast, Ca^2+^ signaling in PSCs has so far been investigated only by a handful of studies. Nevertheless, recent reports indicate that Ca^2+^ signals are also important in the regulation of PSC physiology, showing a clear cross-talk with other pathways, such as NO signaling (Jakubowska et al., 2016). We already know that PSCs express bradykinin receptor type 2 (BDKRB2) (Ferdek et al., 2016), and pharmacological studies revealed that these cells produce Ca^2+^ responses even to very low doses of bradykinin (Won et al., 2011; Gryshchenko et al., 2016a). Since bradykinin is a well-known pro-inflammatory mediator, BDKRB2 signaling may well play a role in activation of PSCs in diseases of the pancreas (Gryshchenko et al., 2016b). PSCs also express a number of Ca^2+^ channels, particularly those from the family of transient receptor potential (TRP). For example, TRPC6 has been associated with autocrine stimulation of PSCs in hypoxic conditions (Nielsen et al., 2017); whereas another TRP member, TRPC1, was proposed to contribute to pressure-induced activation of these cells (Fels et al., 2016). Deregulated Ca^2+^ signals directly underlie the pathophysiology of acute pancreatitis, and thus it is somewhat surprising that so little is known about the effects that common inducers of pancreatic pathology, such as bile acids and ethanol metabolites, have on Ca^2+^ signaling in PSCs. Given that ethanol induces the expression of TRPV4 in these cells (Zhang et al., 2013), whereas sodium cholate and taurocholate generate noxious and sustained Ca^2+^ signals (Ferdek et al., 2016), the contribution of PSCs in the development of acute pancreatitis might still be underrated by the current dogma.

## PSCs in Diseases of the Pancreas

Despite being a minority in the normal pancreas, the role of activated PSCs becomes apparent in pathophysiological conditions. It is well established that persistent activation of PSCs is the main contributor to fibrosis in pancreatic diseases, with pancreatic cancer and pancreatitis being the most prevalent. In health, PSCs regulate ECM turnover not only by producing its components but also by secreting key enzymes engaged in EMC remodeling – matrix metalloproteinases (MMP) as well as their inhibitors – tissue inhibitors of metalloproteinases (TIMPs) (Phillips et al., 2003). PSCs have been shown to activate in response to cytokines upregulated in acute pancreatitis (Mews et al., 2002). Upon activation, the fate of PSCs largely depends on the external stimuli and can follow different scenarios. If the injury is transient, activated PSCs revert to quiescence or become senescent (McCarroll et al., 2003). However, a subpopulation of PSCs may expand excessively when activating factors accumulate or occur in a persistent manner e.g., in chronic pancreatitis (Haber et al., 1999; Masamune et al., 2009). Activated PSCs secrete increased amounts of MMP-2 (Phillips et al., 2003), and this enzyme breaks down normal basement membrane, which then may hasten its replacement by fibril-forming collagen (Friedman, 2000).

Pancreatic cancer – mainly pancreatic ductal adenocarcinoma (PDAC) – remains one of the most serious problems of our modern society. According to the Global Cancer Statistics 2018 (GLOBOCAN 2018), PDAC has been ranked the 11th most frequent cancer worldwide and the 7th most deadly cancer, accounting for 4.5% of all cancer-related deaths (Bray et al., 2018). The hallmark of pancreatic tumorigenesis is a desmoplastic reaction that leads to the formation of fibrotic stroma. The stroma not only provides a hypoxic microenvironment for the neoplastic cells, but it also constitutes a physical barrier that limits the efficacy of drug delivery to the tumor. Despite significant advances in the treatment regimens and surgical procedures, the collagen-rich fibrotic microenvironment of pancreatic cancer is the main reason why chemotherapeutics are largely ineffective and the clinical outcome remains very poor (Dangi-Garimella et al., 2011; Shields et al., 2012). It is becoming increasingly clear that a successful therapy should not only be focused on cancer cells but also target the tumor-associated fibrotic stroma.

Pancreatic stellate cells are the main cellular contributors to the desmoplastic reaction in PDAC. They become progressively activated in the process of tumorigenesis and deposit collagen fibers that embed and protect cancer cells. This collagen-rich microenvironment becomes an integral part of the developing tumor, and the interactions between the stroma and cancer cells are essential practically at every stage of tumorigenesis from its initiation, through progression, and to metastasis. As an example, PSCs have been shown to increase the viability and proliferative capacity of cancer cells as well as reduce gemcitabine-induced apoptosis of these cells. This was attenuated by pharmacological blockade of TGF-β receptor I (TGF-βRI, ALK5) (Liu S.L. et al., 2019). The inhibition of stroma-cancer cell interactions is currently attracting a lot of interest as a promising strategy that may sensitize PDAC to therapy and increase the 5-year survival rate of patients suffering from this disease (Cannon et al., 2018).

## Signaling in PSC-Cancer Cell Interactions

In light of the above, a number of recent studies have been devoted to investigating signaling pathways in tumor–stroma interactions. Yet again, the Hippo pathway stands out as an important player in pancreatic tumorigenesis. YAP1 is expressed by cancer cells as well as is present in the nuclei of PDAC-derived PSCs, correlating with their activated phenotype. Xiao et al. (2019) have recently demonstrated *in vitro* that depletion of YAP1 in PSCs (via silencing its expression) results in a shift of PSC phenotype toward quiescence, evidenced by a downregulation of markers such as α-SMA and collagen I as well as a decrease in PSC contractility and proliferation. While secreted protein acidic and cysteine rich (SPARC) has previously been associated with the poor outcomes of pancreatic cancer patients (Erkan et al., 2008; Whatcott et al., 2015), very recently SPARC has been identified as a downstream target of YAP1 that mediates its effects on cancer cell proliferation via paracrine signaling (Xiao et al., 2019). Since YAP1 expression shows a strong correlation with the degree of tissue fibrosis in patient samples, YAP1 was suggested as a feasible target in PDAC therapy that, in principle, could inactivate PSCs and limit the development of the tumor-associated stroma (Xiao et al., 2019).

Another recently revealed paracrine factor involved in stroma–cancer cell interactions is leukemia inhibitory factor (LIF) (Ohlund et al., 2017; Bressy et al., 2018; Shi et al., 2019). This cytokine is secreted by activated PSCs in PDAC lesions and acts on the neighboring cancer cells via its receptor LIFR, activating the STAT3 pathway, driving tumor progression and increasing chemoresistance (Shi et al., 2019). Since its circulating and tissue levels increase in PDAC patients, LIF was postulated to be both a novel biomarker and a feasible therapeutic target.

Stroma–cellular interaction may also occur via cell surface receptors such as integrins (Schnittert et al., 2018). Integrin α-11 (ITGA11), a collagen type I–binding receptor, is an interesting example: essentially absent in the healthy pancreas, it becomes expressed within the stromal fraction of PDAC tissues. It has been found that approximately 80% of α-SMA-positive cells are also ITGA11-positive, and knockdown of ITGA11 in PSCs results in attenuation of differentiation, migration, and secretion of ECM components by these cells (Schnittert et al., 2019). Another cell surface protein, integrin α-5 (ITGA5), was also shown to be present in as many as 72% of α-SMA-positive cells in human PDAC tissues, and its high expression was associated with poor prognosis (Kuninty et al., 2019). ITGA5 was demonstrated to play a role in TGF-β–mediated activation of PSCs via Smad2 and FAK pathways; and its knockdown inhibited both PSC-induced cancer cell proliferation *in vitro* and tumor growth *in vivo* (Kuninty et al., 2019). The authors went even one step further and developed a novel ITGA5-antagonizing peptidomimetic (AV3) that could inhibit PSC activation and enhance the cytotoxic effects of gemcitabine in spheroid co-cultures of cancer cell lines with PSCs (Kuninty et al., 2019).

Bcl2-associated athanogene 3 (BAG3) is expressed by multiple cancer types, including PDAC, and correlates with poor prognosis. Recent studies show that this marker is also present in activated PSCs and may promote, via IL−6, TGF−β2, and insulin-like growth factor-binding protein 2 (IGFBP2) signaling, autocrine-driven maintenance of the activated phenotype in these cells (Yuan et al., 2019). *Vice versa*, BAG3−positive PSCs also increase migration and invasion capacity of nearby cancer cells via soluble factors such as IL−8, monocyte chemoattractant protein-1 (MCP-1), TGF−β2, and IGFBP2 (Yuan et al., 2019). A recent report shows that plasminogen activator inhibitor-1 (PAI-1), a common regulator of blood coagulation, cell apoptosis, and migration, is secreted by pancreatic cancer cells and activates PSCs LRP-1/ERK/c-JUN signaling (Wang et al., 2019). Interestingly, knockdown of PAI-1 in cancer cells abolished activation in co-cultured PSCs, indicating that PAI-1 might be one of the key players in PSC – cancer cell interaction (Fang et al., 2012).

While cancer-associated PSCs secrete hepatocyte growth factor (HGF), its receptor c-MET is present on pancreatic cancer cells. Simultaneous inhibition of HGF and c-MET combined with gemcitabine is more effective in reduction of tumor volume in an orthotopic model of pancreatic cancer than chemotherapy alone, indicating a critical role of the HGF/c-MET pathway in PSC–cancer cell interactions (Pothula et al., 2017). What is more, inhibition of HGF, c-MET, and urokinase-type plasminogen activator (uPA) has been shown to decrease the angiogenic properties of endothelial cells, suggesting the role of PSCs and the HGF/c-MET pathway in neoangionesis (Patel et al., 2014). The authors envision that a novel antiangiogenic approach that targets the HGF/c-MET and uPA pathways could be used against pancreatic cancer (Patel et al., 2014).

## Mechanosensing and ECM Stiffness

The human pancreas is an organ that weights only approximately 100 g (Innes and Carey, 1994), but it produces daily as much as 1 L (or 1 kg) of pancreatic juice, comprising water, bicarbonate, and a variety of digestive enzymes (Pallagi et al., 2015). This substantial difference between the juice mass and the mass of the tissue is associated with mechanical stress in the organ exerted by the fluid pressure on pancreatic duct walls and pancreatic acini. PSCs have recently been attributed to sensing the mechanical properties of pancreatic microenvironment (Cortes et al., 2019b). PSCs control mechanostasis (mechanical homeostasis of the organ in response to various types of forces) not only in the normal tissue but also under pathophysiological conditions like that present in the development of fibrosis (Ferdek and Jakubowska, 2017). Deposition of collagen fibers by activated PSCs, as well as the presence of cross-linking in these fibers, affects the mechanical properties of the pancreatic microenvironment, making it dense and rigid (Cortes et al., 2019a, b). In light of the recent findings, activated PSCs might be able to sense fiber topology, adhesiveness of their surroundings, and viscoelasticity of the stroma (Papalazarou et al., 2018). Stress, strain, and forces that stretch the fibrillary proteins are also mechanosensed by myofibroblast-like cells (Chen et al., 2017). Importantly, the increased matrix stiffness may further promote activation of PSCs, supporting a positive feedback loop that perpetuates formation of the dense fibrotic tumor stroma (Bachem et al., 2005). Therefore, modulation of the mechanical properties of the desmoplastic pancreatic tissue, in order to decrease its density and overcome problems with drug delivery, may become one of key strategies in successful therapy of pancreatic cancer (Papalazarou et al., 2018). Only very recently has tamoxifen, a drug used successfully against breast cancer cells, been demonstrated to inhibit differentiation of quiescent PSCs into myofibroblasts via the G protein-coupled estrogen receptor (GPER)- and hypoxia-inducible factor-1 alpha (HIF-1α)-mediated mechanism and suppress matrix remodeling (Cortes et al., 2019a, b). Another plausible anti-fibrotic and mechanomodulatory strategy against pancreatic diseases could be targeting YAP and TAZ signal transduction of the Hippo pathway: YAP/TAZ, via their interaction with the cell cytoskeleton, promote cell “stemness,” tissue regeneration, and remodeling of the stroma; whereas YAP can modulate fibroinflammatory responses (Martinez et al., 2019).

What is more, activated PSCs express hyaluronan synthase 2 (HAS2) as well as hyaluronidase 1 (HYAL1) and have been identified as an important source of stromal hyaluronic acid (HA) (Junliang et al., 2019). In turn, HA has been attributed to high interstitial fluid pressure and vascular collapse in PDAC desmoplastic reaction (Provenzano et al., 2012). Enzymatic degradation of HA has been shown to reduce the interstitial pressure, restore the microvasculature and enhance the efficacy of chemotherapy (Kultti et al., 2012; Provenzano et al., 2012; Jacobetz et al., 2013). Of note is that HA binds to CD44, commonly expressed by many types of cancer cells, and this interaction promotes tumor-driving signaling and transport activities (Slomiany et al., 2009; Toole, 2009). Since CD44 has also been found on the surface of PDAC cells (Zhao et al., 2016), the full spectrum of HA roles in pancreatic cancer might be even more complex; but this notion requires further investigation.

## Reversion to Quiescence

Since activation of PSCs can often become part of the pathophysiological process, a number of attempts have been made to either block the phenotype transition in these cells or force already activated PSCs back to quiescence. Treatment with retinol and retinoic acid (both ATRA and 9-RA) was used to inhibit cell proliferation, expression of activation markers and the MAPK signaling pathway in these cells; and retinol even blocked ethanol-induced activation of PSCs (McCarroll et al., 2006). Further, ligands of PPARγ, a nuclear receptor regulating lipid storage and glucose metabolism, inhibited PSC proliferation and decreased expression of α-SMA and MCP-1, suggesting a potential role of PPARγ in the development of pancreatic fibrosis and inflammation (Masamune et al., 2002). More recently, it was shown that vitamin D receptor (VDR) is present in the stroma of human pancreatic tumors, and its ligand, calcipotriol, reduces markers of inflammation and fibrosis in mouse models of pancreatitis and pancreatic cancer (Sherman et al., 2014). A different group demonstrated that while vitamin D2, vitamin D3 and calcipotriol inhibit activation of PSCs *in vitro*, they fail to reverse the phenotype transition after the cells have already been activated (Wallbaum et al., 2018).

## Concluding Remarks

**FIGURE 2:**
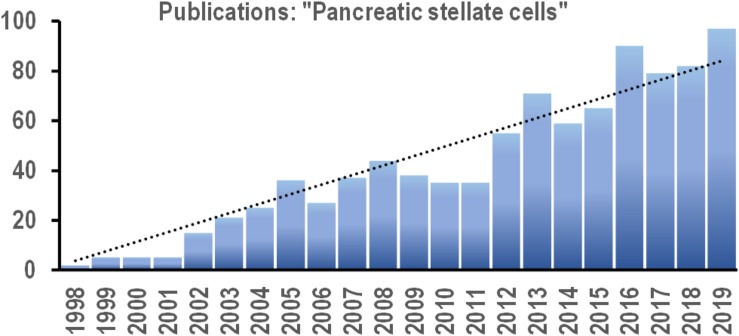
Bar chart shows the number of new scientific papers published each year between 2008 and 2019 with the phrase “pancreatic stellate cells” in the text (based on online search via PubMed.gov). Trend line (dotted): *y* = 3.8179x; *R*^2^ = 0.914.

For the last two decades, there has been a steady (linear) increase in the number of papers on PSCs published every year (Figure 2). This new field continues to expand, and increasingly more efforts are directed toward uncovering the signaling pathways that control PSC physiology. Since PSC activation underlies the pathogenesis of pancreatic disorders, the signals that induce phenotype transition in these cells are of particular interest. Targeted manipulation of these signals might prevent or even revert PSC activation and become a useful tool in the therapy of pancreatic diseases.

## Author Contributions

AK, MS, MJ, and PF contributed to writing the manuscript. PF edited the final version of the manuscript.

## Conflict of Interest

The authors declare that the research was conducted in the absence of any commercial or financial relationships that could be construed as a potential conflict of interest.
